# Physiological traits, gene expression responses, and proteomics of rice varieties varying in heat stress tolerance at the flowering stage

**DOI:** 10.3389/fpls.2024.1489331

**Published:** 2024-12-04

**Authors:** Hui Guo, Wei Tao, Huiyong Gao, Lei Chen, Xiaoyuan Zhong, Maoyan Tang, Guoqing Gao, Tianfeng Liang, Xiaoli Zhang

**Affiliations:** ^1^ Guangxi Key Laboratory of Rice Genetics and Breeding, Rice Research Institute, Guangxi Academy of Agricultural Sciences, Nanning, China; ^2^ Guangxi Academy of Agricultural Sciences, Nanning, China; ^3^ Bureau of Agriculture and Rural Affairs of Xiangfen, Xiangfen, China

**Keywords:** rice, heat stress, physiological, gene expression, proteomics

## Abstract

**Introduction/Background:**

Global warming greatly limits the productivity of rice. Rice plants are highly sensitive to heat stress at the flowering stage. The selection of heat-tolerant varieties is considered the most effective approach for ensuring global food security in the coming decades.

**Methods:**

Based on previous screening and QTL localization results, we selected tolerant varieties (Huang Huazhan, HZ) and susceptible varieties (Yang Dao6, YD) of rice and studied their physiological characteristics, gene expression responses, and proteomic differences of their anthers under heat stress. The differentially expressed proteins (DEPs) were validated by real-time PCR.

**Results:**

The activities of the antioxidant enzymes CAT, SOD, POD, and APX were 8.36%, 9.56%, 20.61%, and 25.34% higher in HZ than in YD under heat stress, respectively. Similarly, the content of proline and soluble sugar was 8.32% and 14.47% higher in HZ than in YD, respectively. The content of MDA and H_2_O_2_ was 8.11% and 39.5% lower in HZ than in YD, respectively. The ratio of endogenous GA_3_/ABA in HZ was 10.65, which was significantly higher than that of YD (3.84). In addition, we validated the candidate genes LOC_Os08g07010 and LOC_Os08g07440 that our team located in 2021, and the result showed that the expression of these two heat-tolerant genes in the anthers was significantly higher in HH than in YH. DEPs involved in the response to heat stress were identified by TMT proteomics, five upregulated and three downregulated differential expression proteins in HH. DEPs were verified by RT-qPCR.

**Discussion:**

These results provide new insights into the physiological characteristics, dominant DEPs, and gene expression responses in both rice varieties under heat stress. Our results indicate that the antioxidant and osmoregulatory capacities, the ratio of endogenous GA_3_ and ABA, these DEPs are mainly involved in the pathways of phenylpropanoid biosynthesis, ubiquitin-mediated proteolysis, carbohydrate metabolism, thiamine metabolism, protein processing in the endoplasmic reticulum, and folding, sorting, and degradation were upregulated to a greater degree in HZ than in YD. Additional studies were performed to clarify the roles of these proteins in response to heat stress.

## 1 Introduction

Rice is a staple crop that provides food for more than half of the world’s population and 60% of the human population in China (He et al., 2023; Yuan, 2014). Climate change, especially increases in temperature driven by rapid economic development and the greenhouse effect, poses major challenges for human society. Heat waves occurred in many countries in the summer of 2022 (Lu et al., 2023), and they have been particularly common in central–eastern China since the 1960s (Ma Y. Y. et al., 2023; Zhang et al., 2023). Rice yield has declined by 10% for each 1°C increase in the nighttime minimum temperature in the dry season (Peng et al., 2004). Under the heat stress, rice yield could decrease by up to 10% in 2030 in South Asia (Lobell et al., 2008) and by 15%–26% by 2080 in developing countries; yields could decrease by 30%–40% in India, and grain losses could reach devastating levels, such as more than 50% in Senegal and Sudan (Cline, 2008). The global human population is rapidly increasing (Rahman and Zhang, 2023). Therefore, warming will have a significant effect on global food security (Driedonks et al., 2016). In light of continuous global warming and the frequent occurrence of high temperatures over short periods, ensuring the sustainable production of rice will require studies of its heat tolerance.

Rice prefers warm temperatures and short periods of sunshine. However, it is sensitive to high temperatures in the booting stage, flowering stage, and grain-filling stage (Jagadish et al., 2007). It is particularly sensitive in the flowering stage given that heat stress over 35°C can result in substantial decreases in pollen fertility and vigor, inhibit anther dehiscence, and lead to poor pollen germination on the stigma (Jagadish et al., 2010; Wang Y. et al., 2019), which affects the grain filling rate and grain yield. Subsequently, it was found that heat stress results in poor rice quality, expressed as reduced palatability, undesirable grain appearance, and increased grain chalkiness at the grain-filling stage (Nevame et al., 2018; Kaneko et al., 2016). The effect of heat stress on rice was directly related to the duration of heat stress, and the higher and longer the heat stress, the more serious the effect on rice (Guo et al., 2010). Therefore, many studies have examined the high-temperature tolerance of rice to mitigate heat damage and losses.

Proteomics studies of rice under heat stress at the flowering stage (Kim et al., 2015; Mu et al., 2017), filling stage (Liao et al., 2014), and milk stage (Timabud et al., 2016) have shown that the differentially expressed proteins (DEPs) vary among stages. These are mainly involved in biosynthesis, energy and metabolism, redox homeostasis, photosynthesis, and signaling, and temporary protective mechanisms play a role in enhancing heat tolerance. Jagadish et al. (2010) first identified 13 DEPs by two-dimensional gel electrophoresis. One cold protein and one heat shock protein (HSP) were found to be significantly upregulated in the heat-tolerant variety N22. Comparison of rice anther protein expression patterns in high-temperature environments and natural environments revealed 139 DEPs, including HSPs, energy metabolism proteins, DnaK family proteins, and chaperonins (Kim et al., 2015). Mu et al. (2017) found that heat stress only induced the degradation of ribosomal proteins in heat-sensitive varieties, and the abundance of HSPs, β-extension proteins, and lipid transfer proteins was increased in heat-tolerant varieties; these changes in protein abundances might be related to differences in high-temperature tolerance. Due to differences in local heat conditions, researchers have studied rice plants under different types of heat stress (stepped temperature gradient and maximum temperature). Compared with the previous studies, the temperature setting was different in the present study, at 38°C for 6 h (from 9:00 a.m. to 3:00 p.m.) lasting 3 days, which was a valid test condition derived from repeated trials locally in Nanning, Guangxi (Liang et al., 2016). In addition, anthers were chosen as the subject, which is distinguished from tissues such as spikes, glume, and leaves in previous studies. Furthermore, tandem mass tags (TMT) (Thompson et al., 2003) has not been frequently used to study the DEPs of rice anthers under heat stress.

In previous studies, we identified various heat-tolerant resources from different rice varieties at the flowering stage from 2012 to 2018 (Wang, Q. et al., 2019) and localized the heat-tolerant QTL *qHTT8* on chromosome 8 in the F2:3 population of the heat-tolerant rice variety Huang Hua Zhan (HZ) and the heat-susceptible rice variety Yangdao6 (YD) in 2021 (Chen et al., 2021). However, the physiological mechanism and the mechanism of protein regulation in the anthers of HZ and YD in response to heat stress remain unclear. We examined the physiological responses, gene expression responses, and proteomic differences in the anthers of HZ and YD under heat stress. The main aim of this study was to clarify the physiological mechanisms underlying the heat tolerance of rice to facilitate the screening of heat-tolerant varieties.

## 2 Materials and methods

### 2.1 Materials and crop husbandry

The experiment was conducted at the Rice Research Institute of Guangxi Academy of Agricultural Sciences (22°51′N, 108°14′ E). The heat stress responses of the rice varieties HZ and YD were determined. The germinated seeds were sown separately in the tray on 20 February and 6 March 2023, and then 30-day-old seedlings were transplanted into plastic pots (35-cm diameter and 35-cm depth). The soil was extracted from rice fields (27.2 g/kg of organic, 2.1 g/kg of total N, 30 mg/kg of total P, 73 mg/kg of total K). Each pot contained 6 kg of dry soil with 10 g of urea (5:3:2 as basal fertilizer, in the tiller stage, and in the spike stage), 5 g of calcium superphosphate (as basal fertilizer), and 10 g of potassium chloride (5:5 as basal fertilizer and in tiller stage). Each variety was planted in 60 pots with three holes per pot and one plant per hole. Both diseases and pests were effectively controlled. Before the flowering stage, the pot was transferred to walk-in growth chambers (4.2 m × 3.2 m × 2.1 m) and treated under different temperatures.

### 2.2 Temperature treatments

Four treatments were performed in this study. Huang Huazhan (HH) and Yangdao6 (YH) were subjected to heat stress, and Huang Huazhan and Yangdao 6 in the control temperature were named as treatments HC and YC, respectively. A total of 240 pots were planted in this experiment. When 2 to 3 cm of the spike of the main tiller was exposed, it was marked with a red string. Half of the pots were randomly moved into the chamber, and high-temperature treatment was performed. Plants were subjected to 38°C ± 0.5°C between 9:30 a.m. and 3:30 p.m. every day for three consecutive days. The other pots were subjected to the control temperature at 28°C. The relative humidity was maintained at approximately 85%, and the light:dark photoperiod was 12-h light/12-h dark. The pots were removed from the chambers at the end of the treatments, and plants were grown naturally until the maturity stage.

### 2.3 Sampling and measurements

#### 2.3.1 Physiological parameters

During treatments, the fresh anthers derived from the middle part of the marked spikes were taken on the third day of the treatments to determine the physiological indexes. Three biological replications were performed for each treatment.

The activities of antioxidant enzymes, including superoxide dismutase (SOD), catalase (CAT), peroxidase (POD), acerbate peroxidase (APX), and the osmoregulatory substance malondialdehyde (MDA), were measured and determined using the method of Li Hesheng (Li et al., 2000). Approximately 0.5 g of fresh anthers was put into a mortar, poured into 5 ml of phosphate buffer with pH 7.8, and ground in an ice bath. The slurry was centrifuged in a freezing centrifuge for 20 min, and the supernatant was poured into a test tube for determination. The colorimetric method of thiobarbituric acid was used to determine MDA content.

The content of osmoregulatory substances including soluble sugar and proline (Pro) was determined using the ninhydrin colorimetric and anthrone colorimetry methods. Determination of soluble sugar content: Weighted 0.1g of fresh anthers into a graduated test tube, added 5–10 ml of distilled water, sealed with plastic film, extracted in boiling water for 30 min (extracted twice), filtered the extract into a 25-ml volumetric flask, repeatedly rinsed the test tube and residue, then set the volume to the scale, and then determined the content of soluble sugar by anthrone colorimetry (Liu et al., 2013). Determination of free proline content: Weighed 0.3 g of fresh rice anthers, put into a test tube with a stopper, added 5 ml of 3% sulfosalicylic acid solution, added the stopper, extracted in a boiling water bath for 10 min, collected the filtrate, and used sulfosalicylic acid extraction, ninhydrin colorimetric assay (Zhang et al., 2009).

Determination of hydrogen peroxide content: Weighted 0.1 g of fresh anthers, added 3 ml of pre-cooled potassium phosphate buffer (PBS, 50 mmol/L, pH 6.5), and ground in an ice bath. The homogenate was centrifuged at 6,000 × *g* for 25 min at 4°C, and the supernatant was the H_2_O_2_ extract. One milliliter of 0.1% titanium tetrachloride (containing 20% H_2_SO_4_) was added. The mixture was centrifuged at 6,000 × *g*, 4°C for 15 min, and the supernatant was used to determine the OD value at 410 nm (Zhang et al., 2009).

The endogenous hormones GA and ABA were extracted and detected using liquid chromatography-tandem mass spectrometry (LC-MS) by Sciex PI400 (DIW of MDS Inc. Toronto, Canada). The detailed process was as follows: the endogenous hormones GA and ABA content: weighted 0.2 g of the sample, added 2 ml of extraction solution, vortexed for 1 min, ultrasonic ice bath for 20 min, placed at −20°C for 16 h, removed the vortex for 1 min in the 3rd and 6th hour, and then removed it in the 16th hour. Centrifuged (4,000 r/min, 10 min), transferred the supernatant, then added 2 ml of the precipitation into the extraction solution, vortexed for 1 min, then sonicated in the ice bath for 10 min, then centrifuged (4,000 r/min, 10 min), combined with the extracted sample, then added 2 ml of the extracted solution, vortexed for 1 min, sonicated on an ice bath for 10 min, centrifuged (4,000 r/min, 10 min), combined the supernatant, added 2 ml of the supernatant into a purification tube containing 0.1 g of adsorbent, vortexed for 2 min, centrifuged (10,000 r/min, 5 min), took 1.5 ml of the supernatant and placed into 2-ml centrifuge tubes, then concentrated under vacuum until the solvent evaporates, and then added 150 µl of 50% solvent into the dry sample. After drying, the sample was re-dissolved in 150 µl of 50% methanol (containing 0.1% formic acid), centrifuged (10,000 r/min, 10 min), and the supernatant was passed through a 0.22-µm organic microporous membrane, vortexed, and mixed, and then detected using liquid chromatography-tandem mass spectrometry.

Proteomics: During treatments, the anthers of labeled spikes were collected on the third day, extracted in centrifuge tubes using a mini-vacuum machine, wrapped in tin foil, placed into liquid nitrogen for quick freezing, and stored at −80°C for spare use; three replications of each treatment were performed. Total anther proteins were extracted (Yang et al., 2011), and the TMT quantitative proteomic method was used to identify the DEPs (Zheng et al., 2022). The RAW data files for protein identification were analyzed using Proteome Discoverer (Thermo Scientific, Version 2.4) against the Uniprot database (https://www.uniprot.org/taxonomy/39946). Physicochemical properties, such as molecular weight, isoelectric point, and total average hydrophobicity of the DEPs, were analyzed using ProtPamm software on the ExPASy website (http://web.expasy.org/protparam/). DEPs were identified using the following criteria: fold change (FC) (>1.5 or <0.67) and p value <0.05. Annotation of all identified proteins, including DEPs, was performed using Gene Ontology (GO) (http://geneontology.org/) and Kyoto Encyclopedia of Genes and Genomes (KEGG) pathway (http://www.genome.jp/kegg/) analyses.

Gene expression: Gene expression patterns were validated using real-time quantitative polymerase chain reaction (RT-qPCR). RNA was extracted using TriZol method assay, then it was reverse transcribed into cDNA used as template for qPCR. The GAPDH gene was used as the reference control in the study. All RT-qPCR reactions were performed using the CFX96 system (DLAB Scientific Co., Ltd., Beijing, China). The real-time PCR reaction mixture was prepared as follows: 1.0 μl of cDNA template, 10 μl of 2× ChamQ Universal SYBR qPCR Master Mix (Q711, Vazyme, China, Nanjing), 0.4 μl of forward primer (10 μM), 0.4 μl of reverse primer (10 μM). Then, ddH2O was added to the PCR reaction mixture with a total volume of 20 μl. The whole real-time PCR procedure included two steps: initial denaturation at 95°C for 30 s, cycling reaction (40 cycles) consisting of annealing at 95°C for 30 s followed by extension at 60°C for 30 s, and the signal acquisition also at cycling reaction.

### 2.4 Statistical analysis

The physiological parameters and gene expression data from RT-qPCR were determined using SPSS Version 13.0 (Lead Technologies, Chicago, Illinois, USA). The multiple comparisons of various treatment and genotype combinations were analyzed using the LSD method of DPS Data Processing System. The DEPs in the statistical analysis were identified using Student’s *t*-test. Origin ver.2024 was used to make graphs.

## 3 Results

### 3.1 Differences in antioxidant enzyme activities of the anthers in different treatments

**Figure 1:**
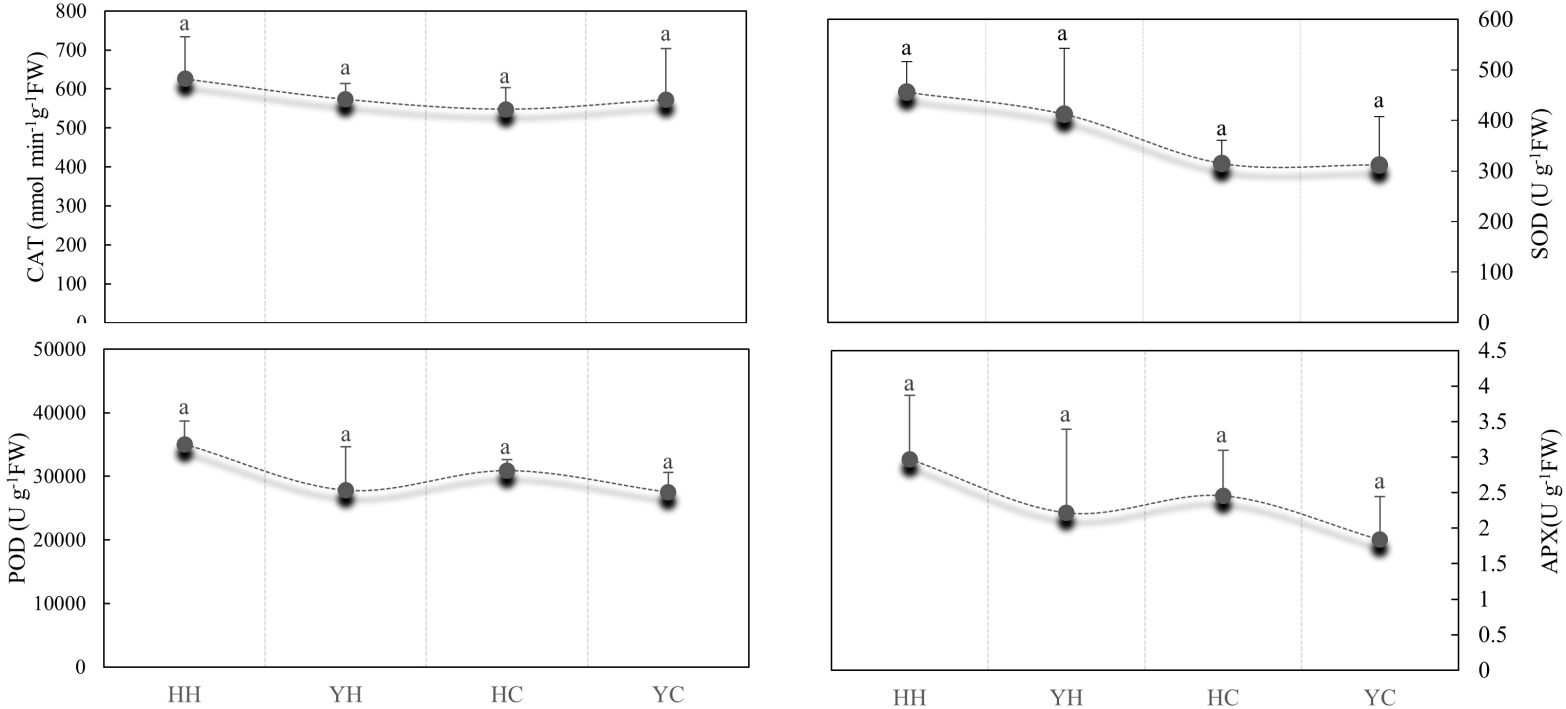
Comparison of differences in the activities of antioxidant enzymes (SOD, POD, CAT, and APX) in anthers from different treatments. Lowercase letters indicate significant differences (p < 0.05) among treatments. Bars show the standard error; the same below.

The effect of antioxidant enzyme activities, such as SOD, POD, CAT, and APX of the anthers, was observed in two rice varieties under HT and CT (**Figure 1**). In HZ, the activities of CAT, SOD, POD and APX were 12.46%, 30.91%, 11.82% and 17.30% compared with CT under HT, whereas the activities of these four enzymes under HT were higher than at CT in YD, by 0.20%, 24.28%, 1.20% and 17.04%, respectively. The difference between HT and CT were higher in HZ than in YD. Under heat stress, the activities of CAT, SOD, POD, and APX were 8.36%, 9.56%, 20.61%, and 25.34% higher in HZ than in YD, respectively, and differences in CAT, SOD, POD, and APX activities were not significant. The activities of the four antioxidant enzymes were significantly higher under heat stress than under control conditions (**Figure 1**). This indicated that the antioxidant capacity of HZ was significantly higher than that of YD.

### 3.2 Differences in the content of osmoregulatory substances and reactive oxygen from different treatments

**Figure 2:**
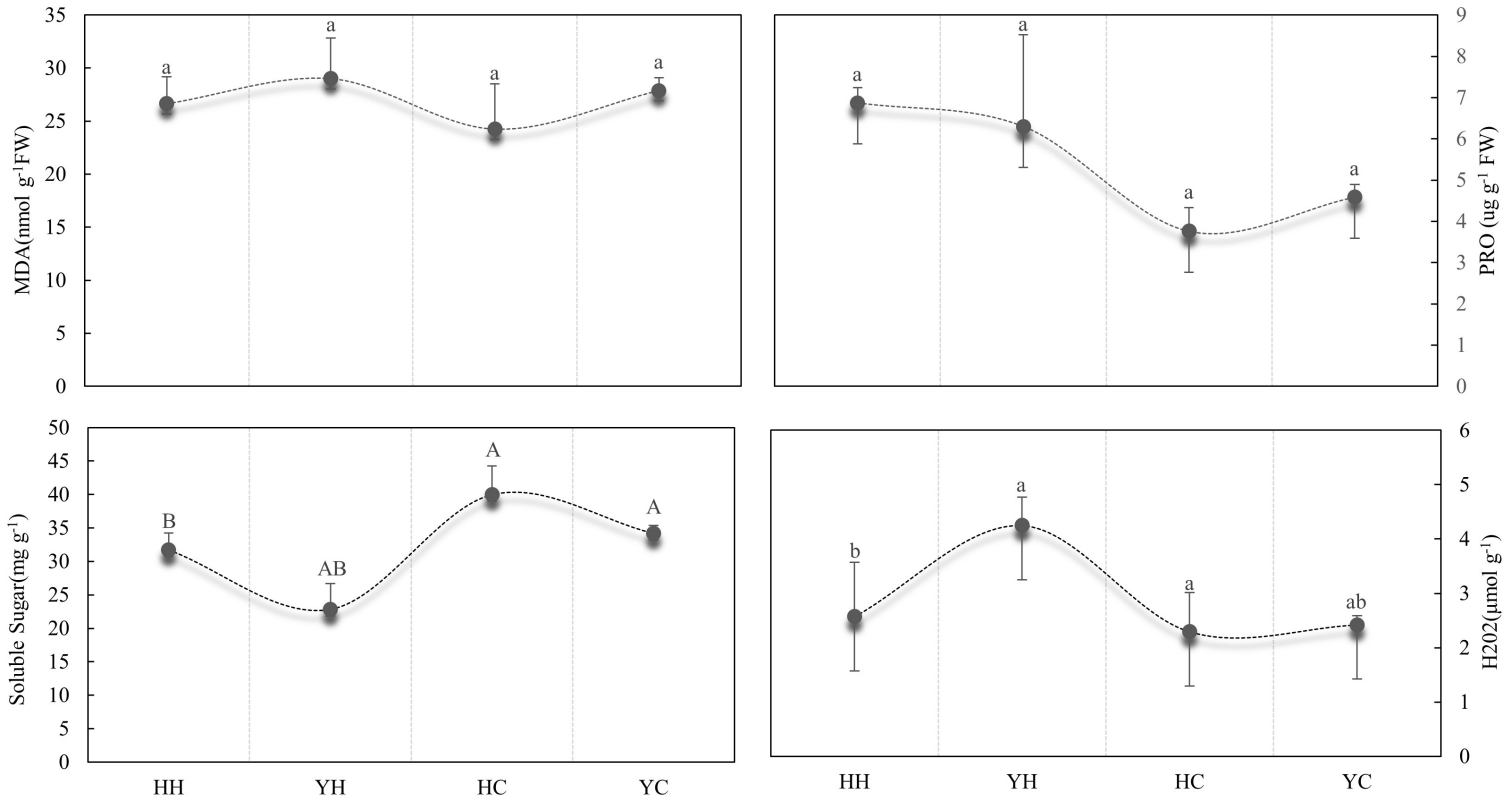
Analysis of the content of MDA, proline, soluble sugar, and H_2_O_2_ in anthers in different treatments. Different lowercase letters and uppercase indicate significant differences (P< 0.05) and extreme significant difference (P< 0.01) among treatments.

Under heat stress, the MDA and H_2_O_2_ content was 8.11% and 39.5% lower in HH than in YH, respectively. The content of proline and soluble sugar was 8.32% and 14.47% higher in HH than in YH, respectively. The content of MDA, Pro, and H_2_O_2_ was higher in the two varieties under heat stress than in control conditions, but the content of soluble sugar declined under heat stress. The membrane peroxidation level and the content of somatically regulated nutrients indicated that membrane damage in the tolerant variety was lower in HZ than in the heat-susceptible variety YD. As for contents of MDA and Pro, the differences among the four treatments were not significant. In contrast, there were highly significant and significant differences on the content of soluble sugars and H_2_O_2_, respectively (**Figure 2**).

### 3.3 Differences in the content of endogenous hormones from different treatments

**Figure 3:**
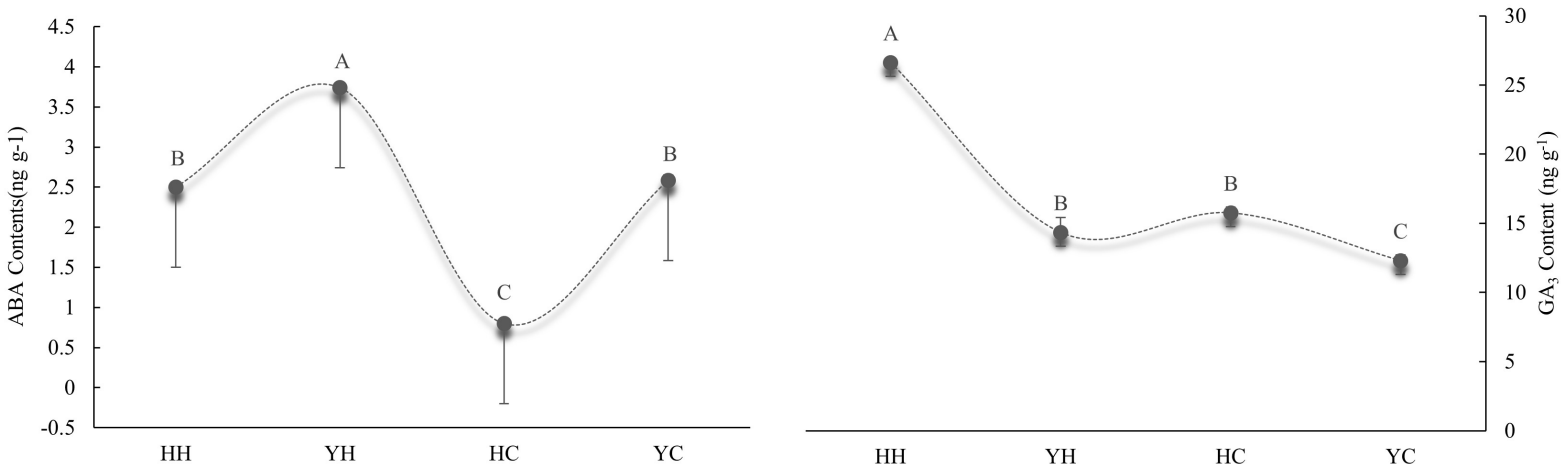
Analysis of the content of endogenous ABA and GA3 in anthers from different treatments and varieties. **(A)** Content of endogenous ABA; **(B)** content of endogenous GA3. Different uppercase letters indicate significant differences (P< 0.01) among treatments.

The content of the endogenous hormones ABA and GA_3_ are shown in **Figure 3**. The ABA content of anthers was higher under heat stress than under the control temperature in both varieties. The ABA content of anthers was higher 33.2% and 69.0% under HT than CT in HZ and YD, respectively. Compared to CT, the content of GA3 in HZ and YD was 40.8% and 14.2% higher under HT,respectively. A similar trend was also observed in the GA content. The GA_3_ content was higher in HZ than in YD under heat stress; by contrast, the ABA content of HZ was lower than that of YD. There was a highly significant level on the content of ABA and GA_3_ (**Figure 3**). The ratio of endogenous GA_3_/ABA in HH was 10.65, which was significantly higher than 3.83 of YH.

### 3.4 Gene expression responses in different treatments

**Figure 4:**
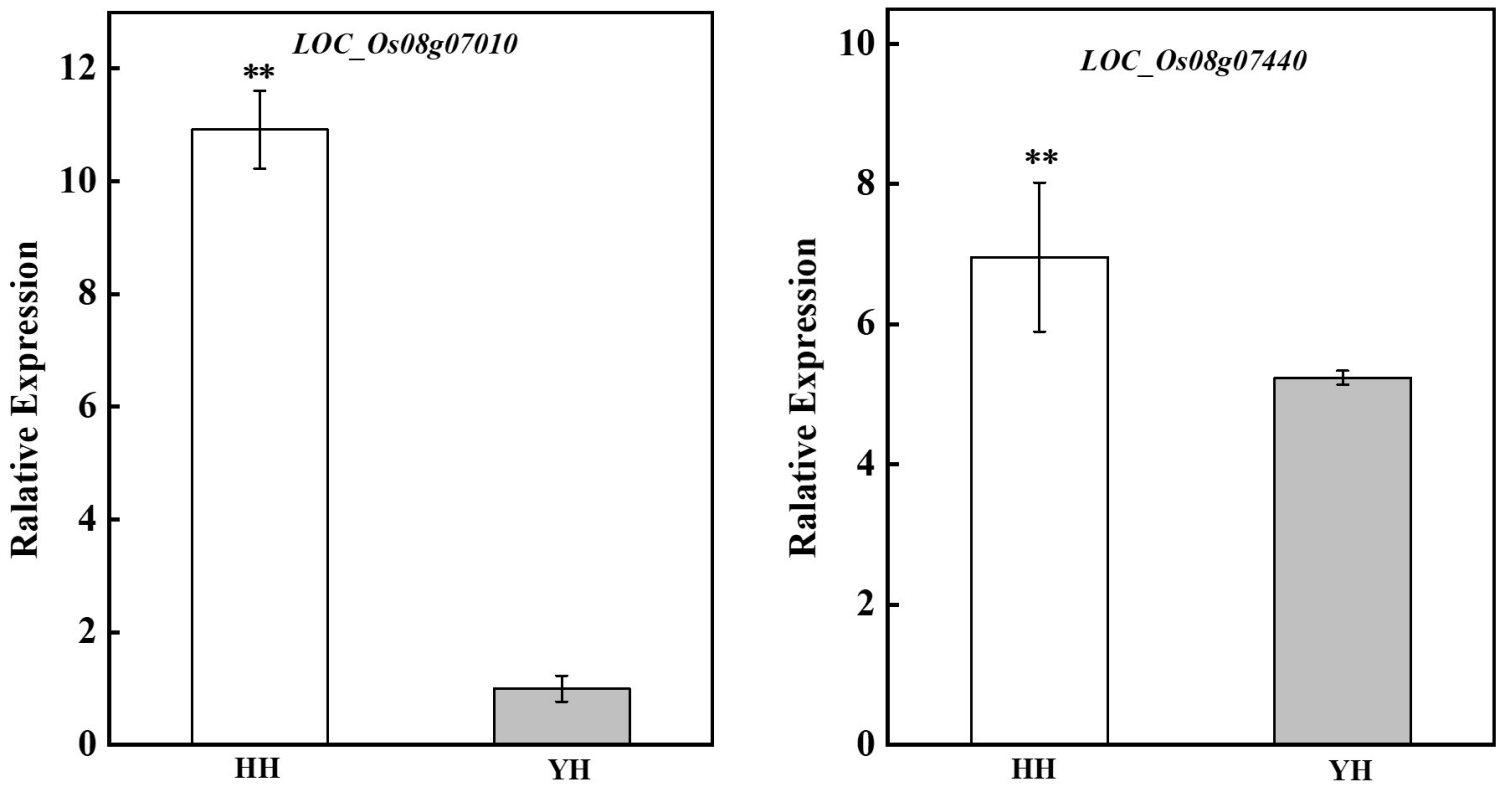
Expression of genes involved in high-temperature resistance in anthers from different treatments and varieties. ** indicates extreme significant difference (P< 0.01).

In the team’s previous study, two heat tolerance candidate genes *LOC_Os08g07010* and *LOC_Os08g07440* were screened by QTL localization using the F2:3 population of Huang Huazhan and 9311 (i.e. YD), which was used for RT-qPCR validation of gene expression levels in the anther under heat stress in the present experiment. The results showed that the expression of these two heat-tolerant genes in the anthers was significantly higher in HZ than YD, which was the same as the validation results using rice spikes (**Figure 4**).

### 3.5 Proteomic differences among treatments

#### 3.5.1 DEPs of varieties under heat stress and the control temperature

**Figure 5:**
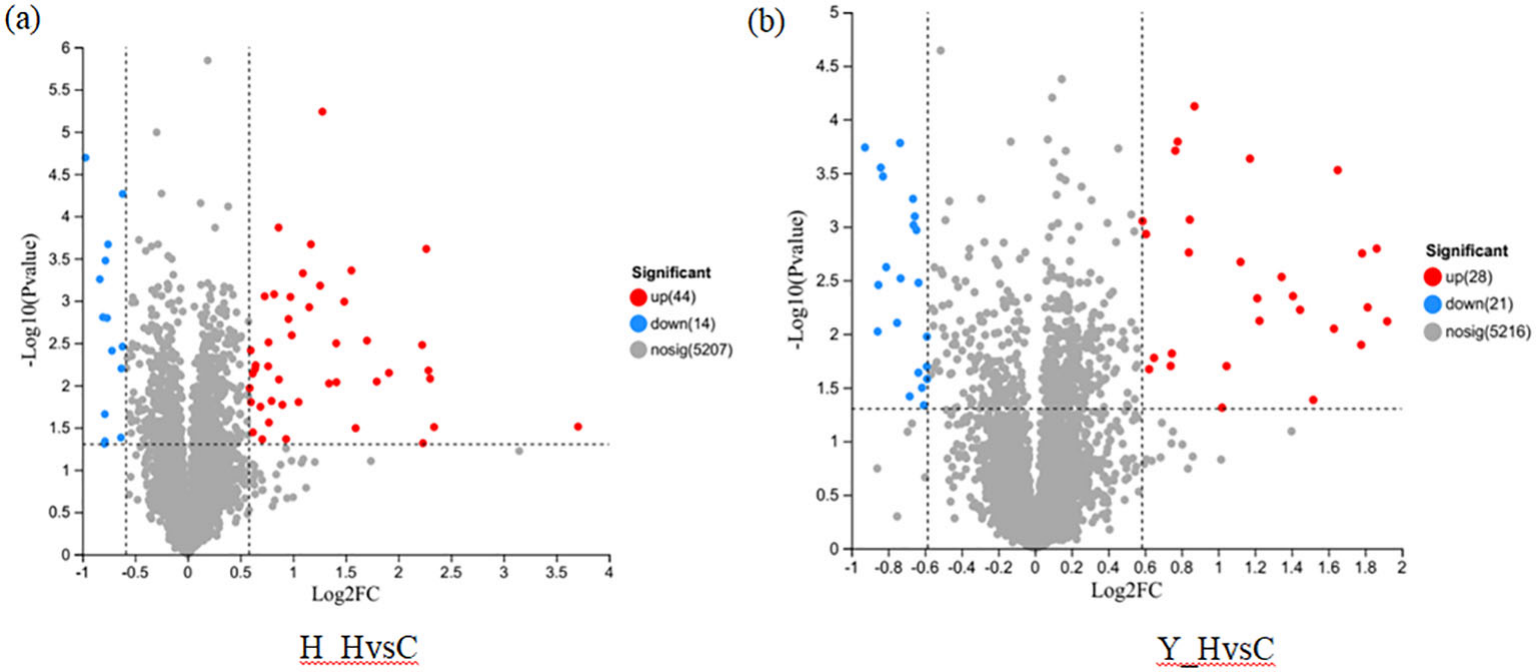
DEPs of different rice varieties under high and check temperatures. **(A)** DEPs in HZ; **(B)** DEPs in YD.

TMT labeling combined with LC-MS/MS technology was used to identify DEPs with the following criteria: FC ≥ 1.5 and p < 0.05. First, DEPs of the same variety under high-temperature stress and control temperature environment were screened. A total of 58 DEPs were identified in HZ, including 44 upregulated proteins and 14 downregulated proteins (**Figure 5A**). A total of 49 DEPs were identified in YD, including 28 upregulated proteins and 21 downregulated proteins (**Figure 5B**).

**Figure 6:**
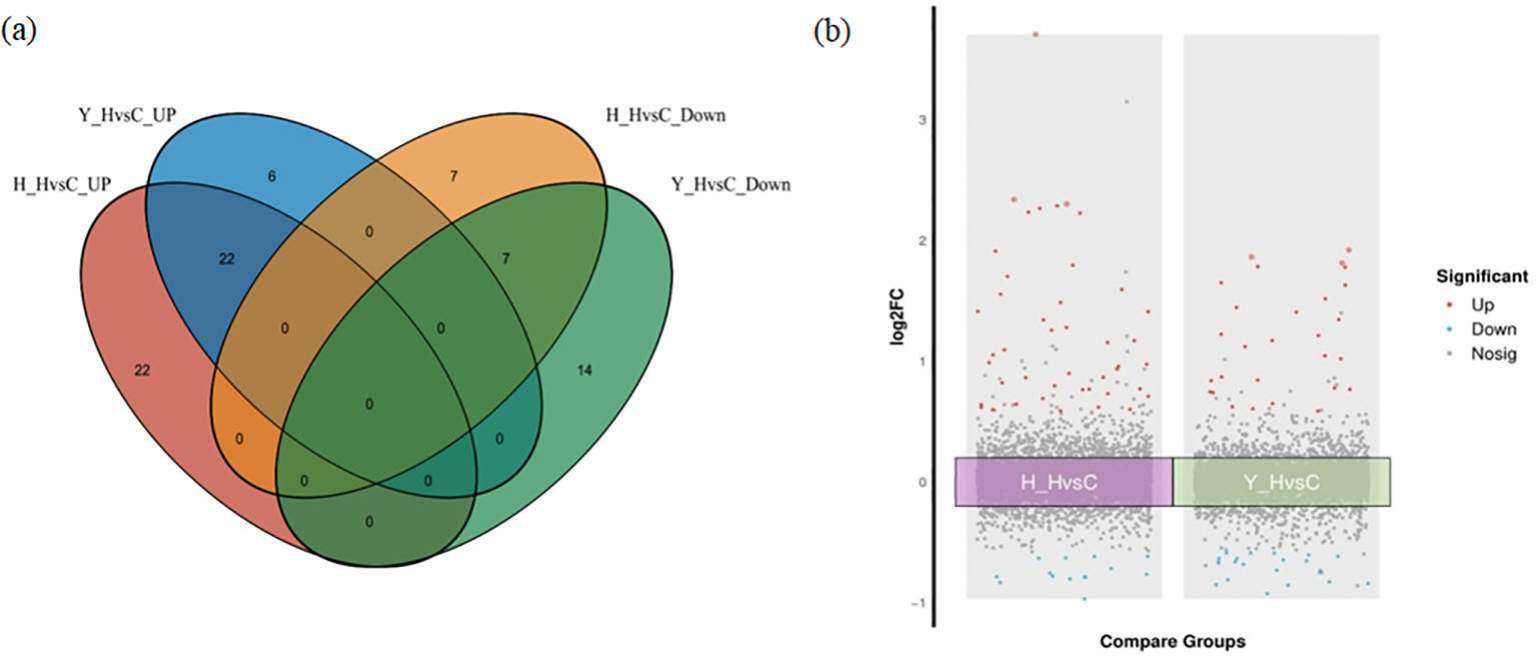
Venn and volcano plots between DEPs of different tolerant rice varieties at high and room temperatures. **(A)** Venn plot of DEPs; **(B)** Volcano plots of DEPs.

According to the DEPs from the two varieties under both heat stress and the control environment, two protein sets were established. DEPs from HZ in the two environments were referred to as H_HvsC. DEPs from YD were referred to as Y_HvsC; DEPs in the two protein sets were subsequently analyzed using a Venn diagram (**Figure 6A**). The results showed that 29 common DEPs with the same expression pattern in the two protein sets were identified, and these included 22 upregulated proteins and 7 downregulated proteins. There were 29 specific DEPs, including 22 upregulated and 7 downregulated proteins, in HZ. In YD, there were 20 specific proteins, including 6 upregulated and 14 downregulated proteins (**Figure 6A**). A volcano plot was made for both the H_HvsC and Y_HvsC protein sets (**Figure 6B**), which indicated that the upregulated DEPs (red dots) and downregulated DEPs (blue dots) located outside the central gray region were DEPs with larger absolute FC values.

#### 3.5.2 GO function annotations of DEPs from two varieties

**Figure 7:**
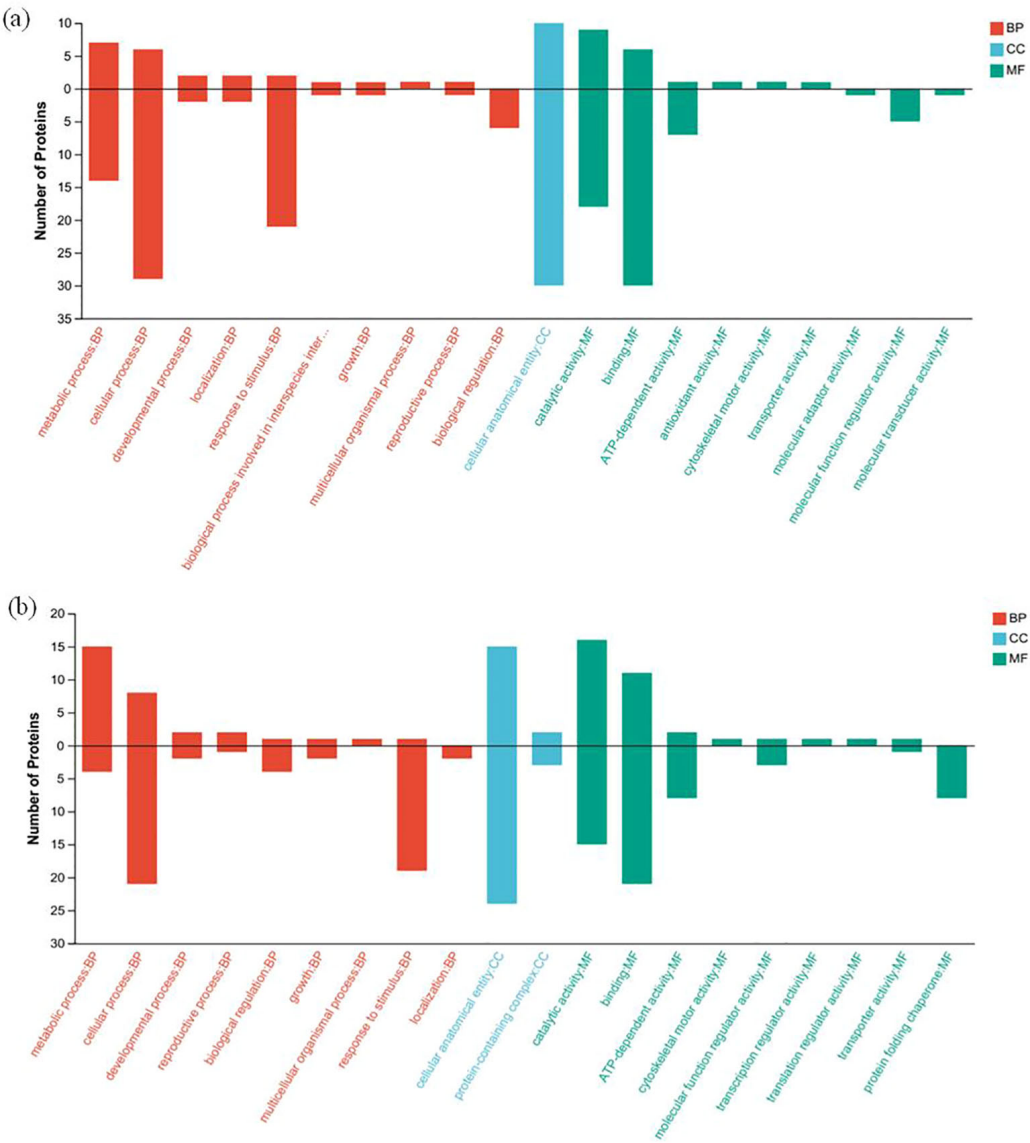
GO annotation analysis of DEPs of rice varieties varying in heat tolerance under high and normal temperature. **(A)** GO annotation of specific DEPs (up- and down-regulated) present in HH; **(B)** GO annotation of specific DEPs (up-regulated and downregulated) present in YD.

**Figure 8:**
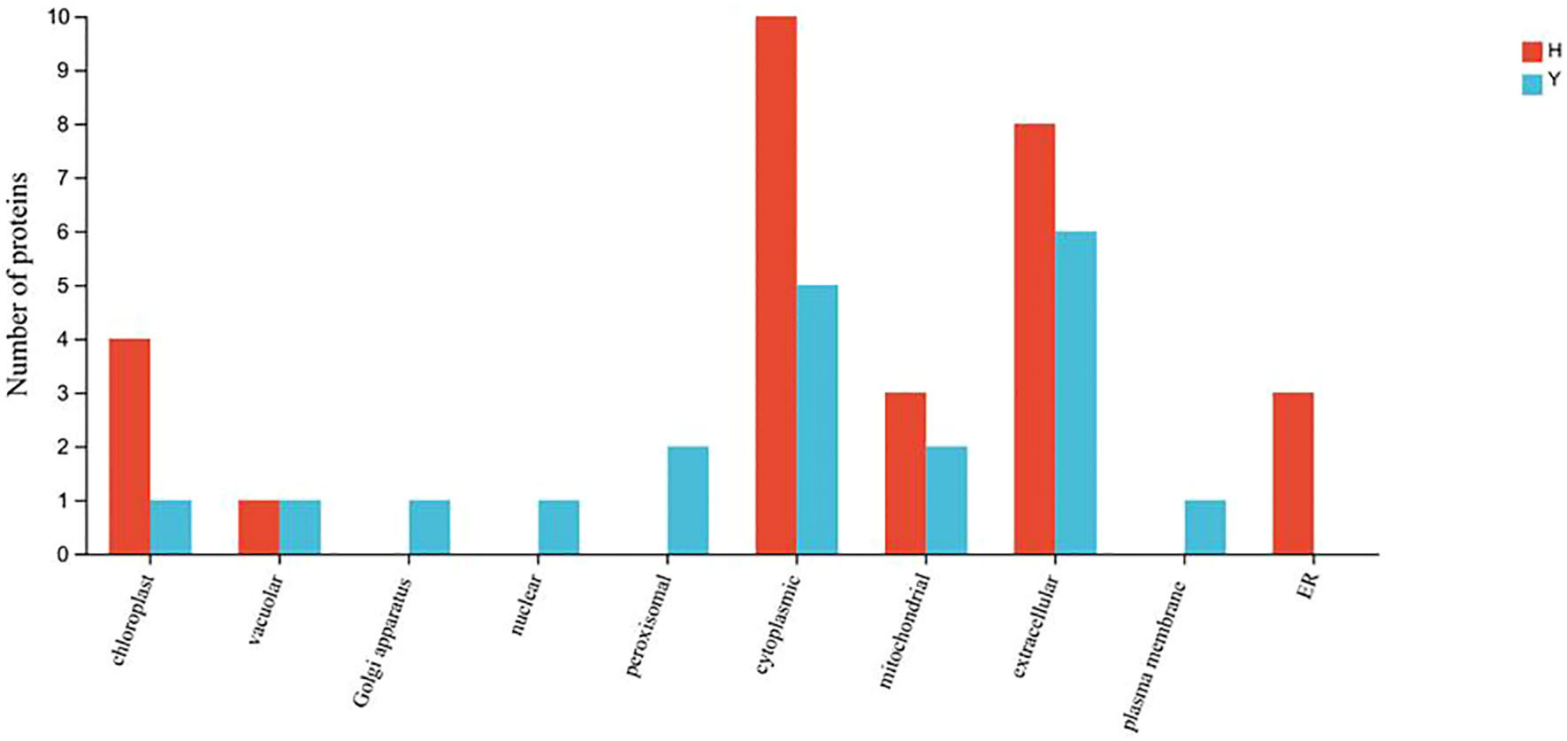
Subcellular localization of specific DEPs in HZ and YD.

Gene Ontology (GO) function annotation analysis was performed for the differentially expressed proteins of HZ and YD, respectively. The 20 most common GO terms were identified for the DEPs of HZ in the biological process (BP) category, and these included cellular process, metabolic process, stimulus response, and biological regulation. The most significantly enriched term in the cellular component (CC) category was cellular anatomical entities. In the molecular function (MF) category, the main GO terms were catalytic activity, bundling, and ATP-dependent activity (**Figure 7A**). It was found that the functions of differentially expressed proteins in YD were basically consistent with those in HZ in BP, CC, and MF (**Figure 7B**). Comparison of subcellular localization databases revealed information on protein subcellular localization and DEP annotations. Multiloc2 software was used to categorize the subcellular localizations of DEPs in HZ and YD (**Figure 8**). The specific proteins of the two varieties were most widely distributed in the cytoplasm, followed by the extracellular matrix, chloroplasts, mitochondria, and endoplasmic reticulum (ER). The DEPs of HH distributed in these subcellular localization were all higher than those in YD. Only the DEPs of YD were localized to the Golgi, nucleus, peroxisome, and plasma membrane.

#### 3.5.3 KEGG pathway enrichment analysis and functional annotations of DEPs from two varieties

**Figure 9:**
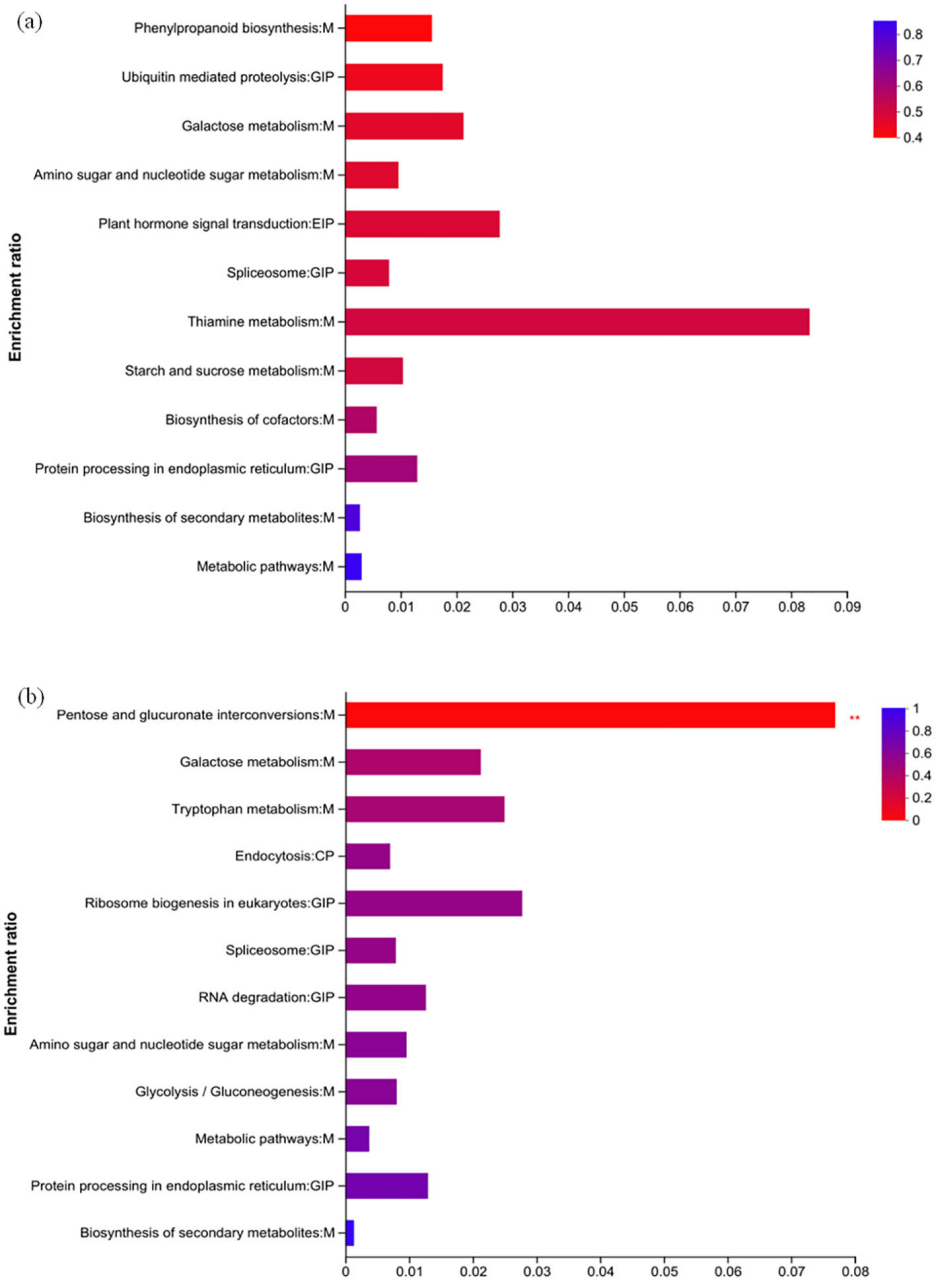
KEGG pathway enrichment analysis of DEPs in two varieties. **(A)** Specific regulated proteins of HH; **(B)** specific regulated proteins of YD.

KEGG pathway enrichment analysis was performed on the DEPs of H_HvsC and Y_HvsC (**Figure 9**). The 20 most significantly enriched KEGG pathways (p < 0.01) were indicated; DEPs in HZ were mainly concentrated in 12 KEGG pathways as follows: phenylpropanone biosynthesis (1), ubiquitin-mediated protein hydrolysis (1), galactose metabolism (1), amino sugar and nucleotide sugar metabolism (1), plant hormone signal transduction (1), spliceosome (1), thiamin metabolism (1), starch and sucrose metabolism (1), biosynthesis of cofactor (1), protein processing in the endoplasmic reticulum (2), biosynthesis of secondary metabolites (2), and metabolic pathways (4) (**Figure 9A**).

KEGG pathway enrichment analysis (p < 0.05) was used to identify DEPs in YD; these accounted for 12 pathways, which include the interconversion of pentose and glucuronic acid (3), galactose metabolism (1), tryptophan metabolism (1), endocytosis (1), ribosome biogenesis in eukaryotes (1), spliceosome (1), RNA degradation (1), amino sugar and nucleotide sugar metabolism (1), glycolysis/glycogenesis (1), metabolic pathways (5), protein processing in the endoplasmic reticulum (2), and biosynthesis of secondary metabolites (1) (**Figure 9B**).

**Figure 10:**
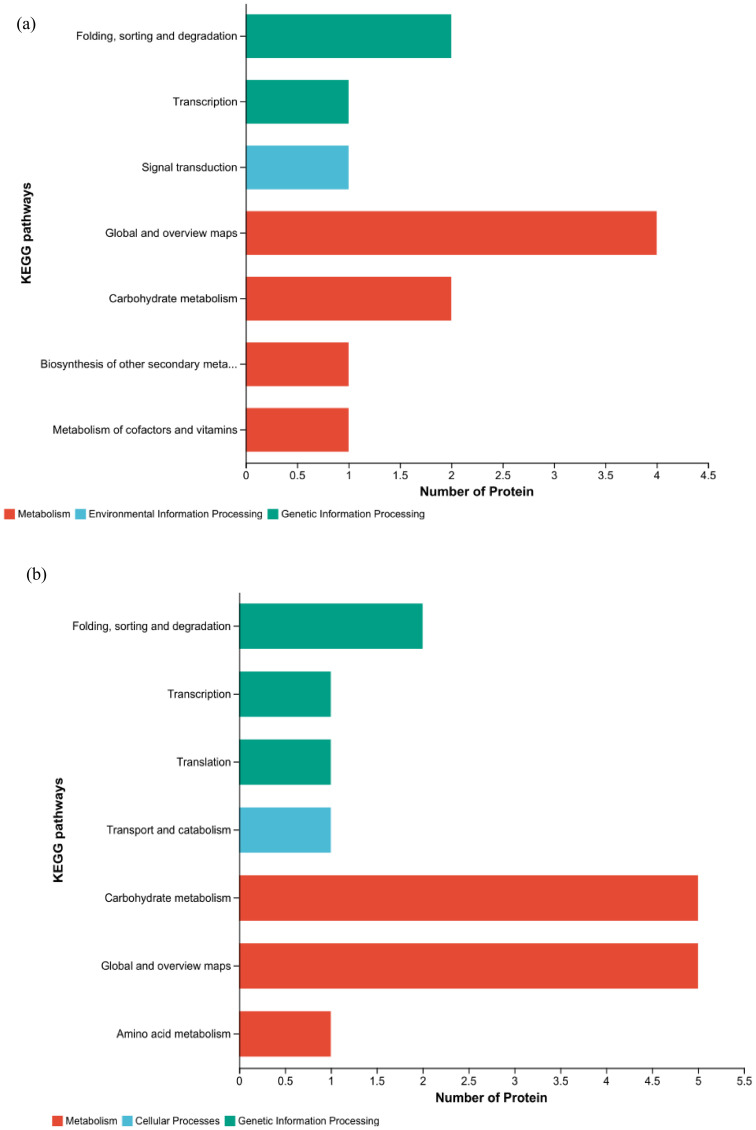
KEGG functional annotations of DEPs in two varieties. **(A)** KEGG analysis of HH; **(B)** KEGG analysis of YD.

KEGG functional annotations analysis was conducted on the proteins to elucidate their functions. In HZ, four DEPs are shown in the metabolic pathway; two DEPs were involved in carbohydrate metabolism, two proteins were involved in secondary material metabolism, and one differential protein was involved in cofactor and vitamin metabolism. In the pathway related to genetic information processing, there was one differential protein each related to folding, sorting and degradation, and transcription; in pathways related to environmental information processing, there was one differential protein involved in signal transduction (**Figure 10A**). In YD, five DEPs were involved in carbohydrate metabolism, one DEP was related to amino acid metabolism, and five DEPs were related to specific function pathways with genetic information processing; one DEP each was associated with folding, sorting and degradation, transcription, and translation. Among the DEPs associated with cellular processes, one DEP was associated with transport and catabolism (**Figure 10B**). The KEGG functional annotations were consistent with the results of the KEGG pathway analysis. Because some DEPs were concatenated in different pathways, eight and nine key DEPs were enriched in different KEGG pathways in HZ and YD, respectively.

## 4 The results of the KEGG pathway and functionally annotated statistics revealed DEPs involved in heat stress tolerance (**Table 1**)

**Figure 11:**
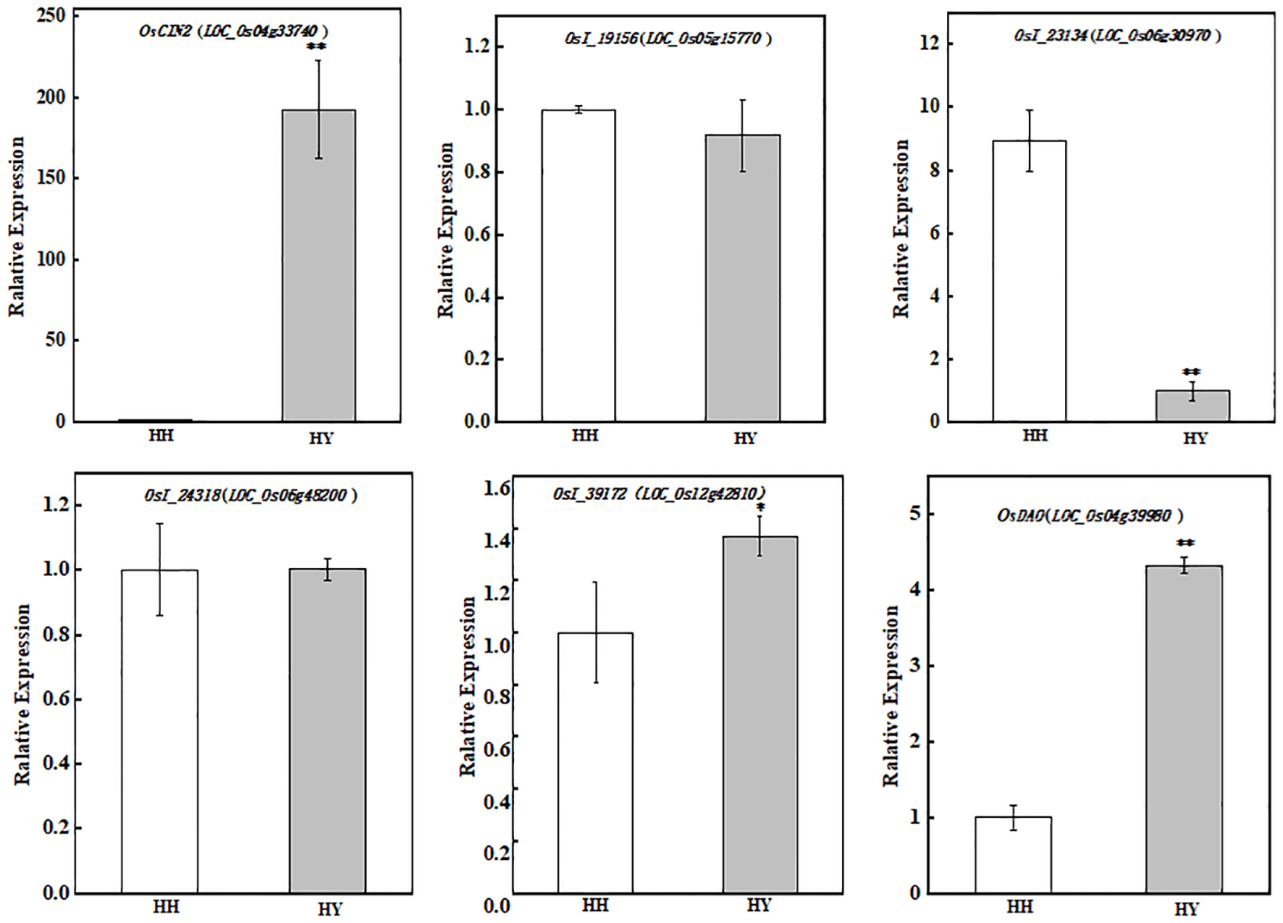
Expression of genes involved in the high-temperature resistance of DEPs from two varieties under heat stress. * indicates significant difference (P< 0.05); ** indicates extreme significant difference (P< 0.01).

The results of the KEGG pathway and functionally annotated statistics revealed DEPs involved in heat stress tolerance (**Table 1**). Six representative DEPs with functions in metabolism, genetic information, and environmental information processing were validated, including Q01IS7 (LOC_Os04g33740) and A2Y2C4 (LOC_Os05g15770), which are related to carbohydrate metabolism; A2YDE3 (LOC_Os06g30970), which is involved in folding, as well as sorting and degradation; A2YGL2 (LOC_Os06g48200), which is involved in signal transduction; B8BN36 (LOC_Os12g42810), which plays a role in translation; and Q01IX6 (LOC_Os04g39980), which plays a role in amino acid metabolism. The consistency of the expression patterns inferred via RT-qPCR with the proteomic data indicated that our data were robust (**Figure 11**).

**Table 1:** Expression model and functional illustration of the DEPs between HH and YD.

Variety	Accession	Protein	Description	Pathway category	FC	Regulate	Coverage %	MW (kDa)
Huang Huazhan	Metabolites							
	A2YCW1	OsI_22951	Peroxidase	Biosynthesis of other secondary metabolites;	0.646*	Down	58	40.6
	Q01IS7	CIN2	Beta-fructofuranosidase, insoluble isoenzyme 2	Carbohydrate metabolism	0.608*	Down	42	66.2
	A2Y2C4	OsI_19156	GH18 domain-containing protein	Carbohydrate metabolism	5.064*	Up	24	32.5
	A2YM28	THI1	Thiamine thiazole synthase, chloroplastic	Metabolism of cofactors and vitamins	1.866*	Up	23	37
	Genetic information processing							
	B8B881	OsI_25296	Thioredoxin-like protein YLS8	Transcription	1.984**	Up	10	16.5
	A2YDE3	OsI_23134	UBC core domain-containing protein	Folding, sorting, and degradation	1.82**	Up	7	16.4
	A2WKD2	OsI_00282	SHSP domain-containing protein	Folding, sorting, and degradation	2.663**	Up	4	16.6
	Environmental information processing							
	A2YGL2	OsI_24318	Xyloglucan endotransglucosylase/hydrolase	Signal transduction	0.652*	Down	11	32.4
Yang Dao6	Metabolites							
	A0A679B8Z3	K0161H03	Putative beta xylosidase	Carbohydrate metabolism	0.563**	Down	19	88.6
	A2XHE1	OsI_11823	Aldose 1-epimerase	Carbohydrate metabolism	0.665*	Down	15	40.7
	A2YQ68	OsI_27427	Pectinesterase	Carbohydrate metabolism	0.635**	Down	32	62.1
	B8BMI9	OsI_38743	Pectinesterase	Carbohydrate metabolism	0.554**	Down	33	44.4
	B8B4K7	OsI_23684	Uncharacterized protein	Carbohydrate metabolism	0.644**	Down	39	43.6
	Q01IX6	DAO	2-Oxoglutarate-dependent dioxygenase DAO	Amino acid metabolism	0.632**	Down	21	32.1
	Genetic information processing							
	B8BN36	OsI_39172	COP9 signalosome complex subunit 6	Translation	0.601**	Down	2	56.1
	A2XEW6	OsI_10881	Uncharacterized protein	Folding, sorting, and degradation	2.652**	Up	32	17.3
	Cellular processes							
	B8BD19	OsI_31865	Uncharacterized protein	Folding, sorting, and degradation; transcription	2.176**	Up	31	70.9

* P-value<0.5, significant difference; **P-value <0.01, extreme significant difference.

## 4 Discussion

The successful identification of heat-resistant quantitative-trait loci (QTL) revealed significant differences in the response to heat stress between HH and YD (Chen et al., 2021). Physiological, gene expression, and proteomic analyses of these two varieties were conducted to elucidate the regulatory mechanisms underlying the heat stress tolerance of rice, which ensures the robustness of our results. Plants do not remain passive when exposed to heat stress; they can adapt to heat stress and rapidly perceive stimuli, generate and transduce signals, and activate antioxidant enzyme systems, which accelerate the removal of ROS and maintain ROS homeostasis (Hasanuzzaman et al., 2013; Zafar et al., 2020). Thus, improving the high-temperature tolerance and effects of the antioxidant system of rice depends on the heat-tolerance capacity of each variety (Visakh et al., 2024). Under heat stress, increases in the activity of the antioxidant enzymes SOD, CAT, POD, and APX were greater in the plants of heat-tolerant varieties at the flowering stage than in heat-sensitive varieties, which is consistent with the results of previous studies (El-Remaly, 2023; Yang et al., 2022b). The content of soluble sugars and proline was higher in HH than in YD, which promoted osmoregulation and the structural stability of cell membranes after exposure to high-temperature stress. In addition, it has been shown that MDA was able to inhibit the activity of cytoprotective enzymes and reduce the content of antioxidants, which aggravated the peroxidation of membrane lipids (Xu et al., 2021), and the results of this study showed that HZ produced significantly less MDA than YD under heat stress. This indicates that anther cell membrane disruption and electrolyte permeation were more severe in YD than in HZ under heat stress, which led to a reduction in its physiological functions and thus the lack of resistance to heat stress. This is also one of the reasons why HZ is more resistant to high temperature than YD. In terms of the same variety, the antioxidant enzyme activity of anthers was increased under high stress compared to control temperature, and the results were similar to those of Zhang et al. (2014), but different from some studies (Tang et al., 2008; Zhao et al., 2018); it was caused by the difference in sampling time. The activity of antioxidant enzyme increased, and reached the peak on the third day. With the prolongation of high temperature treatment, the antioxidant enzyme activity were hurt and decreased (Zhang et al., 2014).

When plants are subjected to environmental stress, the content of ABA increases sharply, and there is a significant positive correlation between the accumulation of ABA and the enhancement of stress tolerance (Dar et al., 2017). This is also the reason why the ABA content of HH was higher under heat stress than under normal temperature. Excessive ABA inhibits the expression of sucrose-converting enzymes and monosaccharide transporter protein genes, which leads to the accumulation of a large amount of sugar in anthers, including sucrose. Given that sucrose cannot be transported to pollen, this leads to a reduction in rice pollen fertility and the fruiting rate (Ji et al., 2011). In our study, the spikelet fertility of YD was 14.3%, which was lower than 54.5% of HZ under high-temperature stress (Chen et al., 2021), which was probably caused by the accumulation of a higher amount of ABA in the anthers in the HZ. A physiological and biochemical response is often regulated not only by a single hormone but by multiple hormones and their balance with each other. GA is also key under stressful environments. High-temperature stress increased ABA content but decreased the content of active cytokinin (CK), gibberellin A_1_ (GA_1_), and indole-3-acetic acid (IAA). These factors reduced rice grain weight (Wu et al., 2016). Due to enhanced biosynthesis, reduced degradation, or release of bound forms, ABA concentrations increase under high stress (Chen et al., 2022). It induced a significant increase in ROS and ABA content in developing the anther of rice at the spike stage under high-temperature stress, and ABA regulated PCD and microspore apoptosis in anther chorioallantois cells by “triggering” ROS production, which leads to the formation of high-temperature pollen abortion (Zhao et al., 2023). In the present study, the content of endogenous ABA and GA3 was increased under heat stress. The difference of GA_3_ may be caused by the different types of GA measured and varieties. The ratio of GA_3_/ABA content is more convincing; the ratio of endogenous GA3/ABA in HZ was 10.65 under heat stress, which was significantly higher than that in YD (3.84), and responses in hormone levels ultimately enhance reproductive performance.

A number of rice heat tolerance genes, including transcription factors that are induced by exposure to heat, that are expressed in the floral organs were identified. In 2021, our team screened two candidate genes LOC_Os08g07010 and LOC_Os08g07440 by spikelets and QTL (Chen et al., 2021). In the present study, anthers were used for RT-qPCR validation under the same high-temperature treatment, and the results were consistent with the results of previous studies that LOC_Os08g07010 and LOC_Os08g07440 were highly induced in HZ compared with those in YD under heat stress. Both candidate genes were expressed in spikelets, glumes, and anthers (Zhao et al., 2019; https://rice.uga.edu/). However, according to the NCBI and UniProt databases, the proteins encoded by the genes in the BSA region did not intersect with the differential proteins in this study. The candidate genes in the BSA interval may be involved in the regulation of resistance to high temperature, but there is no significant difference in the protein level, or the protein expression is so low that it could not be detected. Furthermore, we homologated the protein sequences encoded by the genes in the BSA interval and found the proteins expressed in this study, of which seven had a similarity greater than 80% (**Supplementary Table S1**). Based on gene function annotation, we focused on three genes. LOC_Os08g06430 encodes mitochondrial NADH ubiquinone oxidoreductase, which is involved in rice environmental stresses such as stress tolerance under compartmentalized stress (Chatterjee et al., 2024) and salt tolerance (He et al., 2024). LOC_Os08g07760 (OsBAK1) encodes a kinase for the BR signaling receptor BRI1, which has important roles in rice growth and development and stress tolerance (Chen et al., 2014; Song et al., 2022). LOC_Os08g06550 encodes acyl-coenzyme A-binding proteins, a class of lipid transport proteins, which have a high binding capacity for acyl-coenzyme A and phospholipids and play an important role in plant growth and development and stress response process (Xu et al., 2019).

TMT proteomics has been widely applied because of its higher quantitative accuracy, fewer missing values, and higher efficiency compared with other methods of proteomic analyses (Creskey et al., 2023). Previous proteomics studies of the response to heat stress have identified proteins involved in heat shock, energy metabolism, DnaK family proteins and chaperonins (Jagadish et al., 2007), DOF family, HSPs, ROS-related proteins (Zhang et al., 2019), HSPs, β-expansins, and lipid transfer proteins (Mu et al., 2017). In this study, we found that the identified heat-resistant proteins also contained energy metabolism and HSP, which was consistent with previous studies. Through GO annotation, we found that the classification functions annotated by DEPs of both HH and YD were basically the same at the CC, MF, and BP levels in the present study. Concurrently, it showed the pathways enriched in HH and YD partially overlaps and some difference by KEGG pathway. Overlapping functions included carbohydrate metabolism, folding, sorting and degradation, transcription, and biosynthesis of secondary metabolites. We identified new upregulated proteins involved in HSPs, UBC core domain-containing protein, thioredoxin-like proteins (Trxs), thiamine thiazole synthase (THI1), beta-fructofuranosidase (BFru), and xyloglucan endotransglycosylase/hydrolases (XTH) in HH. In YD, We founded that amino acid metabolism, carbohydrate metabolism, and COP9 signalosome (CSN) were downregulated. We queried the literature and found that the differential protein accessions A2WKD2, Q01IS7, A2YDE3, A2YM28, B8B881, A2XEW6, and B8BD19 in this study corresponded to the coding genes LOC_Os01g04340, LOC_Os04g33740, LOC_Os06g30970, LOC_ Os07g34570, LOC_Os10g34520, LOC_Os10g34520, LOC_Os03g16020, and LOC_Os09g31486, which were also recognized as differential genes in high temperature stress transcriptome analysis (Vitoriano and Calixto, 2021; Wilkins et al., 2016; Wang et al., 2020).

HSPs were verified to play a role in the response to heat stress by preventing the irreversible aggregation of denatured proteins (Sarkar et al., 2009; Wang et al., 2004; Yang et al., 2022a). HSPs are one of the major types of molecular chaperone proteins identified to date. They are usually induced by exposure to high temperatures, contribute to the correct folding of specific proteins, and assist in *trans*-membrane translocation during plant stress responses. According to the former study, it showed that high molecular mass Hsps include Hsp70, Hsp90, and Hsp100, and small heat shock proteins (sHsps) include Hsp20 (Yang et al., 2022a). This study identified that sHsps domain-containing protein were upregulated in the heat-tolerant variety HZ. This suggested that sHsps played a role in the regulation of heat resistance which was congruent with that of the present study (Jagadish et al., 2007; Zhang et al., 2019; Mu et al., 2017). Concurrenly, We found that the expression of UBC core domain-containing proteins was upregulated in HZ, similar to sHsps, under heat stress (**Table 1**). Endoplasmic reticulum-associated degradation (ERAD) is one of the pathways maintaining the homeostasis of the endoplasmic reticulum (ER), and UBC has a major effect on the stability of certain ERAD substrates under certain types of stress (Cui et al., 2012; Ma, W. et al., 2023). In addition, UBC enzymes play indispensable roles in the biological processes of plants, such as plant growth and stress responses (Jue et al., 2015). Although HSPs have been thought to be solely responsible for the response to heat stress (Umarani et al., 2020), other proteins, including ubiquitin, cytosolic Cu/Zn-SOD, and Mn-POD, are also expressed following exposure to heat stress indicating that they are also involved in the response to heat stress. In algae, the content of the ubiquitin protein complex increases under heat shock (Li et al., 2014). The present study identified COP9 signaling associated with ubiquitination. The initial discovery of the COP9 signalosome (CSN) complex was made via a genetic analysis of light control in *Arabidopsis* seedling development. CSN itself is a target of kinase activity and regulates the activity of kinases via the entire ubiquitin degradation pathway, which affects the biological functions associated with target proteins (Orit Harari-Steinberg, 2004). It was found that CSN plays an important regulatory role in plants in response to both heat and cold stress (Qin et al., 2020). This study’s conclusion was the same. In addition to its interaction with E3s, CSN can regulate protein hydrolysis by binding to protein kinases and deubiquitinating enzymes (Schwechheimer, 2004). In this study, the activity of CSN was reduced under high temperatures in YD, and it may be one of the reasons why its heat resistance is relatively poor.

Thioredoxin-like proteins (Trxs) are mainly involved in the splicing and transcription of mRNAs according to the Uniprot database. In previous studies, Trxs have been shown to play an important role in chloroplast metabolic pathways (Kang et al., 2019). Trxs play key roles in maintaining the function of the b6f complex, which drives ATP synthesis, maintains the energy balance, and maintains redox homeostasis in chloroplasts (Chen et al., 2024). Trx is involved in the light-induced activation of key enzymes in the Calvin Cycle to improve photosynthetic efficiency (Da Fonseca-Pereira et al., 2021). The Trx/Prx/Srx system is involved in plant signaling under stress, especially under abiotic stress (Sevilla et al., 2015), and these signals are important cues that affect plant yield and growth. Trx and Trx-like proteins can regulate chloroplast function by controlling the redox state of various photosynthesis-associated proteins in *Arabidopsis* (Yokochi et al., 2021). In the present study, TRX was also upregulated in HH suggesting that it is mainly from photosynthesis and chloroplasts that heat tolerance is improved. Similarly, thiamine thiazole synthase (THI1) is also expressed as an upregulated protein. THI1 is involved in abscisic acid (ABA) signal transduction, stomatal closure in guard cells, and in the response to drought stress in *Arabidopsis thaliana* (Li et al., 2016). Stomata also affect photosynthesis to some extent, thereby increasing resistance to abiotic adversity. Thiamine plays an important role as an enzyme cofactor in glycolysis, the pentose phosphate pathway, and the tricarboxylic acid cycle. It can regulate cell tolerance to DNA damage, and it is activated after plants are attacked by pathogens. The abundance of THI1 protein has been shown to increase under heat stress in previous studies (Xu and Huang, 2018; Zhang et al., 2013), which suggests that it might be involved in DNA protection or repair. In our study, THI1 regulates the metabolism of cofactors and vitamins to provide more energies to resist heat stress; the expression of THI1 was also upregulated in the heat-tolerant variety HZ under heat stress (**Table 1**).

Heat stress can interfere with carbohydrate metabolism in rice, disrupt the energy balance, and inhibit the antioxidant capacity and heat stress protein accumulation, which lead to decreases in yield and quality. The expression of enzymes involved in carbohydrate interconversion, including putative beta xylosidase, aldose 1-epimerase, and pectinesterase, was downregulated in YD compared with that in HZ under heat stress, which indicates that energy deficiency leads to reductions in the heat tolerance of rice. Similarly, the expression of beta-fructofuranosidase, insoluble isoenzyme 2 (BFru), which is involved in carbohydrate metabolism, was also downregulated. BFru mediates the degradation of sucrose into glucose and fructose, which is the first step in starch biosynthesis (Zhang et al., 2024); the glucose and fructose products then enter the glucuronate pathway, which ultimately produces energy and carbon dioxide. This is attributed to the expression of proteins that mediate decreases in energy metabolism under high-temperature stress. This indicates that high temperatures interfere with energy metabolism and severely inhibit the growth and development of organisms.

DEPs involved in environmental information processing were identified. For example, xyloglucan endotransglycosylase/hydrolases (XTH) can disrupt and reconnect xyloglucan chains and modify the structure of cellulose–xyloglucan complexes to rebuild the cell wall. XTH plays a critical role in the aluminum (Al) tolerance of tea trees (*Camellia sinensis*) (Wu et al., 2021). XTH plays a role in determining the structure and composition of plant cell walls. Therefore, elucidating changes in XTHs during the response of plants to abiotic stress is important for studying plant cell wall signaling-mediated stress regulation mechanisms in plants (Chen et al., 2024). The TaXTH gene regulates the drought response in wheat. The expression of TaXTH12.5a enhances the drought tolerance of *Arabidopsis thaliana* (Han et al., 2023). In this study, XTH is related to signal transduction in heat-tolerant rice varieties; in subsequent studies, we will investigate its signaling role in regulating heat-tolerant functions.

In summary, we found that the proteins associated with heat tolerance mainly included HSPs proteins, carbohydrate metabolism, maintain endoplasmic reticulum stability, photosynthetic system, cell wall signaling, and are ubiquitination-related by TMT. Through the KEGG pathway analysis, these DEPs were found to be mainly involved in the pathways of phenylpropanoid biosynthesis, ubiquitin-mediated proteolysis, carbohydrate metabolism, thiamine metabolism, and protein processing in the endoplasmic reticulum in this study.

We analyzed the physiological characteristics, expression of the heat tolerance genes, the candidate genes LOC_Os08g07010 and LOC_Os08g07440 that our team located in 2021 under heat stress, and protein expression patterns in heat-tolerant and susceptible varieties of rice via TMT proteomics. We found that the antioxidant capacity was stronger, the content of osmoregulatory substances and the GA/ABA ratio were higher, and DEPs were mainly involved in the pathways of phenylpropanoid biosynthesis, ubiquitin-mediated proteolysis, carbohydrate metabolism, thiamine metabolism, and protein processing in the endoplasmic reticulum. Folding, sorting, and degradation were upregulated to a greater degree in HZ than in YD, all of which likely contribute to the greater heat tolerance of HZ compared with that of YD. Additional studies are needed to clarify the associations of these proteins with heat stress regulation through signaling.

## Data Availability

The datasets presented in this study can be found in online repositories. The names of the repository/repositories and accession number(s) can be found below: https://www.iprox.cn/, IPX0009330001.
