# Supplementary material for: Physiological traits, gene expression responses, and proteomics of rice varieties varying in heat stress tolerance at the flowering stage
Source: Front Plant Sci. 2024 Dec 4;15:1489331. doi: 10.3389/fpls.2024.1489331 (PMC11656201; doi:10.3389/fpls.2024.1489331)
Supplement: Supplementary file 1 [file Table1.docx]

| Supplementary Table1 Results of homology comparison of Protein encoded by genes in the BSA interval | | | |
| --- | --- | --- | --- |
| Accession | MSU_ID | Identity (%) | MSU_annotation |
| B8BAX7 | LOC_Os08g06430 | 100 | mitochondrial NADH-ubiquinone oxidoreductase, putative, expressed |
| B8BB64 | LOC_Os08g07490 | 100 | expressed protein |
| B8BB68 | LOC_Os08g07760 | 99.84 | BRASSINOSTEROID INSENSITIVE 1-associated receptor kinase 1 precursor, putative, expressed |
| A2YRP3 | LOC_Os08g07500 | 98.824 | DUF617 domain containing protein, expressed |
| B8B7D9 | LOC_Os08g06880 | 90.741 | expressed protein |
| A6N0A7 | LOC_Os08g06610 | 87.234 | mps one binder kinase activator-like 1A, putative, expressed |
| A2Y8J9 | LOC_Os08g06550 | 80.247 | acyl CoA binding protein, putative, expressed |
